# Distinct Methodologies to Produce Capped Mesoporous Silica with Hydroxyapatite and the Influence in Intracellular Signaling as Cytotoxicity on Human Umbilical Vein Endothelial Cells

**DOI:** 10.3390/bioengineering8090125

**Published:** 2021-09-12

**Authors:** Aline Oliveira da Silva de Barros, Luciana Magalhães Rebêlo Alencar, Frank Alexis, Ralph Santos-Oliveira

**Affiliations:** 1 Laboratory of Synthesis of Novel Radiopharmaceuticals and Nanoradiopharmacy, Nuclear Engineering Institute, Brazilian Nuclear Energy Commission, Rio de Janeiro 21941906, Brazil; alinedbcg@gmail.com; 2 Laboratory of Biophysics and Nanosystems, Department of Physics, Federal University of Maranhão, São Luís 65080805, Brazil; lucianamagal@gmail.com; 3 School of Biological Sciences and Engineering, Yachay Tech University, San Miguel de Urcuquí 100119, Ecuador; falexis@yachaytech.edu.ec; 4 Laboratory of Radiopharmacy and Nanoradiopharmaceuticals, Zona Oeste State University, Rio de Janeiro 23070200, Brazil

**Keywords:** hydroxyapatite, mesoporous silica, biological, cytotoxicity

## Abstract

Mesoporous silica has unique properties such as controllable mesoporous structure and size, good biocompatibility, high specific surface area, and large pore volume. For that reason, this material has been broadly functionalized for biomedical applications, such as optical imaging, magnetic resonance imaging (MRI), positron emission tomography (PET), computed tomography (CT), ultrasound imaging, and widely employed as drug delivery systems. In this study, we synthesized fiber-type mesoporous silica capped with hydroxyapatite (ordered SiO_2_–CaO–P_2_O_5_ mesoporous silica). Its biological activity was evaluated through a cellular and molecular approach using HUVEC cell culture. Two distinct methodologies have produced the ordered SiO_2_–CaO–P_2_O_5_ mesoporous silica: (i) two-step Ca-doped silica matrix followed by hydroxyapatite crystallization inside the Ca-doped silica matrix and (ii) one-step Ca-doped silica matrix formed with the hydroxyapatite crystallization. Further analysis included: elemental analysis, transmission, scanning electron microscopy images, Small and Wide-Angle X-ray Diffraction analysis, Fourier Transform Infrared, and in vitro assays with HUVEC (cytotoxicity and immunoblotting). The hydroxyapatite capping methodology significantly affected the original mesoporous material structure. Furthermore, no cellular or molecular effect has been observed. The promising results presented here suggest that the one-step method to obtain hydroxyapatite capped mesoporous silica was effective, also demonstrating that this material has potential in biomedical applications.

## 1. Introduction

Mesoporous silica particles are a promising system due to their highly ordered and stable structure permeated by mesopores separated by 1 to 4 nm walls. This system presents a large surface area (greater than 1000 m^2^ g^−1^), a large number of pores in sizes (from 2 to 15 nm), and varied volumes (greater than 1 mL g^−1^) [1,2,3,4,5,6]. Moreover, they have good biocompatibility and bioactivity [7,8,9]. The physicochemical properties of mesoporous particles could affect their circulation time and biodistribution, including the shape, size, and surface functionalization [5,6]. The literature has reported that mesoporous particles with varied shapes typically accumulate into the liver, lungs, and spleen [10,11]. Moreover, their physicochemical properties also affect their interactions with hepatocytes and Kupffer cells [12,13,14,15,16]. Several coating strategies have been developed during or after the synthesis method to reduce the particle accumulation into the liver and the clearance time [17,18,19]. The presence of hydroxyapatite (HAP) in mesoporous silica is associated with increased bioactivity, biocompatibility, and non-inflammatory behavior [20]. Additionally, the presence of HAP in the mesoporous silica structure increases the drug loading capability and biodegradability [21].

Although mesoporous silica particles may be used for delivery of drugs, proteins, and genes [22], for the treatment of tumors [23], imaging [24,25,26], tissue engineering [27], and photodynamic therapy [28]. Some significant challenges remain associated with toxicity, pharmacokinetics, and biodistribution [29,30]. The expanded use of mesoporous silica in biomedical applications relies on translation as the biological safety aspects of these particles. Pasqua et al. [31] have demonstrated the safety aspect of mesoporous silica in cells. However, Pinto et al. [32] have shown that mesoporous silica can cross the transplacental barrier and reach the fetus with a high bio-accumulation in the brain. Chen et al. [33] stated that mesoporous silica might cause systemic inflammation in vivo, impair vascular homeostasis and alter vascular reactivity. Controversially, Bhavsar, Patel, and Sawant [34] have demonstrated that mesoporous silica is unable to cause cytotoxicity or cause hemolysis in HEK-293 cells even at high doses (200 µg/mL). Additionally, they found mesoporous silica safe for i.v. use in vivo at a dose up to 40 µg/kg.

The lack of consensus about the safety aspect of mesoporous silica, especially the capped one, motivates data acquisition about the interaction of this system with biological ones. This study synthesized, characterized, and tested the biocompatibility (in vitro) of HAP in capped ordered SiO_2_–CaO–P_2_O_5_ mesoporous silica.

## 2. Materials and Methods

### 2.1. Synthesis of Hydroxyapatite-Coated Mesoporous Silica Particles

#### 2.1.1. Method 1—SBA-15/HAP-1

The material synthesis has been carried out using a two-step Ca-doped silica matrix, followed by hydroxyapatite (HAP) crystallization inside the Ca-doped silica matrix, as reported by Diaz et al. [35]. The first step (low pH step) consisted of the preparation of a calcium doped matrix. Pluronic-123 block copolymer (Sigma-Aldrich, Saint Louis, MO, USA) (EO_20_PO_70_EO_20_; molecular weight 5750 g/mol) was used as a structure-directing agent and Tetraethyl orthosilicate (TEOS) (Sigma-Aldrich, Saint Louis, MO, USA), anhydrous CaCl_2_ (Sigma-Aldrich, Saint Louis, MO, USA) as silica and calcium sources, respectively. Briefly, copolymer P-123 was dissolved under agitation in a solution containing deionized water, HCl (Sigma-Aldrich, Saint Louis, MO, USA) 2 M, and CaCl_2_. After complete dissolution of the copolymer P-123, TEOS was slowly added to the solution under stirring at 40 °C. After 24 h under stirring, the gel was hydrothermalized at 80 °C for 24 h. Then, the solid was dried in the oven.

In the second step (high pH step), the calcium-doped silica matrix was added under stirring into a solution containing deionized water and Na_2_HPO_4_·2H_2_O (Sigma-Aldrich, Saint Louis, MO, USA). The solution pH was adjusted to 9–10 with NH_4_PO_4_ (Sigma-Aldrich, Saint Louis, MO, USA), and the stirring was maintained. After 2 h under stirring, the solution was submitted to a second hydrothermal treatment at 80 °C for 24 h. Then, the solid was washed and calcined at 500 °C for 10 h. The final molar composition was: 1 TEOS:3.1 HCl:114 H_2_O:0.012 P-123:1 Ca:1 P.

#### 2.1.2. Method 2—SBA-15/HAP-2

The material synthesis has been carried out using a one-step Ca-doped silica matrix formed with the HAP crystallization, as reported by Diaz et al. [35]. Two stock solutions (0.29 M) were prepared: CaCl_2_ (S1) and Na_2_HPO_4_·2H_2_O (S2). In a typical synthesis, calcined SBA-15 was added to deionized water. Then, under stirring, solution S1 was dropped into the SBA-15 suspension. After homogenization, solution S2 was slowly added. The pH was adjusted to 9–10 with NH_4_PO_4_ solution. After 24 h, the product was washed and dried. The final molar composition was: 1 TEOS:3.1 HCl:114 H_2_O:0.012 P-123:1 Ca:1 P.

### 2.2. Elemental Analysis (EA)

The determination of the contents of Silicon (Si), Calcium (Ca), and Phosphorus (P) was performed using the Inductively Coupled Plasma Optical Emission Spectrometry (ICP-OES) technique using a Perkin Elmer optical emission spectrometer, model Optima 4300 DV.

### 2.3. Scanning Electron Microscopy (SEM) Analysis

The morphology of mesoporous particles and their hydroxyapatite coating was examined by Scanning Electron Microscopy (SEM) (JEOL LSM 5800). The samples were sputter-coated with a layer of gold for observation at 10 kV.

### 2.4. Transmission Electron Microscopy (TEM) Analysis

The mesoporous structures of particles and their hydroxyapatite coating were examined by Transmission Electron Microscopy (SEM). Micrographs were recorded using a JEOL transmission electron microscope (TEM) model JEM-2010 with a LaB6 filament as the electron source, operated at 200 kV. Material samples were mounted on a microgrid carbon polymer, supported on a copper grid, by placing a few droplets of a suspension of the sample in water followed by drying at the ambient temperature.

### 2.5. Small-Angle X-ray Diffraction (SXRD) and Wide-Angle X-ray Diffraction (WXRD) Analysis

Small-Angle X-ray diffraction was performed in Siemens D5000 Kristalloflex equipment using a Ni filter and CuKα radiation source (λ = 0.15406 nm) operating at a voltage of 40 kV and a current of 40 mA. Scans were performed in the 2*θ* range from 0 to 10 with a count time of 3 s with 0.02° intervals. The Wide-Angle X-ray Diffraction was performed using X’Pert Pro Panalytical equipment in the range of 2*θ* from 10 to 80 with CuKα radiation source (λ = 0.15406 nm).

### 2.6. Fourier Transform Infrared (FTIR) Analysis

The FTIR spectrum of all the samples was recorded on Nicolet Nexus 470 FT-IR spectrometer to identify functional chemical groups and bonds, using a wavelength scattering from 450 to 4000 cm^−1^. The samples were grounded with KBr in a 2% *w*/*w* proportion and pressed into thin wafers using a Caver press of 7 tons.

### 2.7. Biological Characterization

#### 2.7.1. In Vitro Studies

Once the mesoporous silica SBA-15/HAP_2 showed a better mesoscopic formation, we decided to evaluate this nanoparticle in primary cell lines to check its influence on molecular and cellular levels of these nanoparticles. In this direction, we assessed the SBA-15/HAP_2 in the following cell lines: NGM (Human melanocyte cell line) (Cell Bank of Rio de Janeiro, Rio de Janeiro, Brazil), FGH (Human gingival fibroblast cell line) (Cell Bank of Rio de Janeiro, Rio de Janeiro, Brazil), and HUVEC (Human umbilical vein endothelial cells).

#### 2.7.2. Cell Culture

##### Human Melanocytes

Human melanocytes (NGM) cell lines were obtained from the Cell Bank of Rio de Janeiro, Rio de Janeiro, Brazil. They were maintained in DMEM medium supplemented with 10% FBS, NaHCO_3_ (3.7 g/L), HEPES (5.2 g/L), and penicillin (0.5 U/mL). Then, the cells were incubated at 37 °C in a humidified atmosphere of 5% CO_2_ and grown into 75 cm^2^ culture flasks until they reached the confluence when they were detached by rapid treatment with trypsin (0.1%)/EDTA (0.01%).

##### Human Fibroblast

Human fibroblast (FGH) cell lines were obtained from the Cell Bank of Rio de Janeiro, Rio de Janeiro, Brazil. The cells were maintained DMEM/F12 medium supplemented with 10% FBS, NaHCO_3_ (3.7 g/L), HEPES (5.2 g/L), and streptomycin (0.5 mg/mL). Then, the cells were incubated at 37 °C in a humidified atmosphere of 5% CO_2_ and grown into 75 cm^2^ culture flasks until they reached the confluence when they were detached by rapid treatment with trypsin (0.1%)/EDTA (0.01%).

##### Human Endothelial Cells

A modification of the procedure for obtained human umbilical vein endothelial cells (HUVECs) previously described by Jaffe et al. (1973) [36] has been used to produce our own HUVEC cells. In this regard, the HUVEC cells were grown in 199 medium (M199, Sigma Aldrich, St. Loius, MO, USA) supplemented with 20% Fetal bovine serum (FBS, Cultilab Campinas, São Paulo, Brazil), penicillin (0.5 U/mL), and streptomycin (0.5 mg/mL). Endothelial cells were used at passage 3.

#### 2.7.3. Proliferation Assay

Human cells (NGM, FGH, and HUVEC), at a concentration of 5 × 10^3^ cells/well, were seeded in 96-well plates and attached for 24 h. Then, cells were treated in the presence or absence of nanoparticle SBA-15/HAP_2 (20 µg/mL) for another 24 h, followed by washing. The number of attached cells was determined using the MTT assay, as described by [37]. All the analyses were done in triplicate.

### 2.8. Intracellular Signaling—Immunoblotting

Human cells (NGM, FGH, and HUVEC) at a concentration of 2 × 10^5^ cells/well were seeded in 24-well plates and allowed to attach for 24 h. Then, cells were treated in the presence or absence of nanoparticle SBA-15/HAP_2 (20 µg/mL) for another 24 h, followed by washing. The total protein concentration of the samples was determined using the BCA assay according to the manufacturer’s protocol. In this direction, cell lysates were boiled for 5 min with buffer (50 mM Tris–HCl (Sigma Aldrich, St. Loius, MO, USA) pH 6.8, 1% SDS, 5% 2-mercaptoethanol (Sigma Aldrich, St. Loius, MO, USA), 10% glycerol (Sigma Aldrich, St. Loius, MO, USA) 0.001% bromophenol blue (Sigma Aldrich, St. Loius, MO, USA)) for denaturation. The denatured samples (20 µg total protein) were analyzed by immunoblotting analysis. The molecular weight of the separated bands was done by the Rainbow TM protein molecular weight markers. Additionally, the samples were analyzed with the following primary antibodies: anti-p-ERK (1:1000) (Thermo Fisher Scientific, Walthan, MA, USA), anti-ERK (1:1000) (Thermo Fisher Scientific, Walthan, MA, USA), anti-p-Akt (1:1000) (Thermo Fisher Scientific, Walthan, MA, USA), and anti- Akt (1:1000) (Thermo Fisher Scientific, Walthan, MA, USA); anti-α-tubulin (1:1000) was from Sigma Aldrich, St. Loius, MO, USA), anti-GAPDH (1:1000) (Invitrogen, Walthan, MA, USA). The secondary antibodies against rabbit IgG and mouse IgG from Dako (1:2000) (Dako-Agilent Technologies, Santa Clara, (California), USA. For this, the membranes were blocked with 5% BSA (Sigma Aldrich, St. Loius, MO, USA) diluted in TBS (Sigma Aldrich, St. Loius, MO, USA) containing 0.5% Tween-20. All analyses were done in triplicate.

### 2.9. Statistical Analysis

For multiple comparisons, we used one-way ANOVA, followed by Bonferroni post-test analyses. For comparisons between the two groups, we used an unpaired t-test. Differences between groups with *p* < 0.05 were considered statistically significant. Studies were carried out using the GraphPad Prism 8 software for Windows.

## 3. Results and Discussion

### 3.1. Synthesis and Characterization of Hydroxyapatite-Coated Mesoporous Silica Nanoparticles

**Figure 1:**
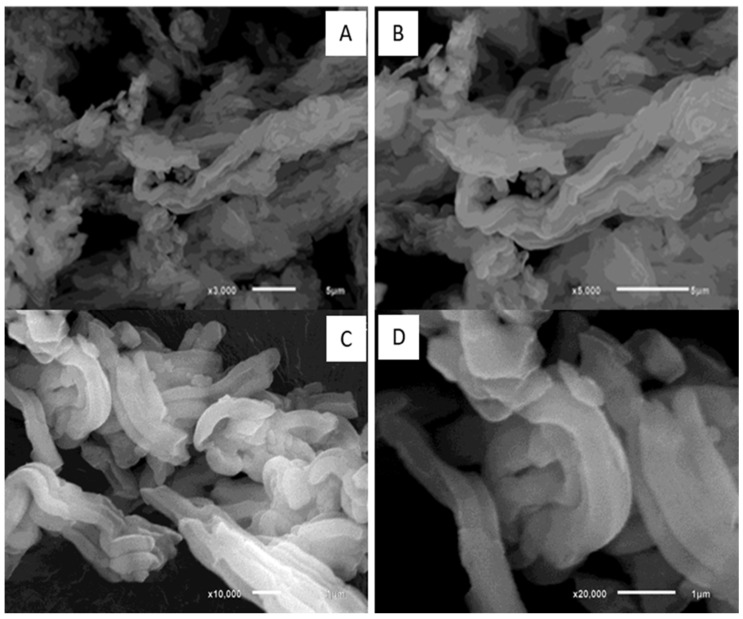
Scanning Electron Microscopy images of the SBA-15 mesoporous layered material and its aggregates at different magnifications (**A**–**D**).

The first material synthesis (SBA-15/HAP_1) has been carried out using a two-step Ca-doped silica matrix, followed by hydroxyapatite (HAP) crystallization inside of the Ca-doped silica matrix, as reported by Diaz et al. [35]. The second synthesis process (SBA-15/HAP_2) has been carried out using a one-step Ca-doped silica matrix formed with the HAP crystallization, also according to Diaz et al. [35]. The incorporation of hydroxyapatite (HA) into mesoporous silica by different methodologies showed that these approaches resulted in two distinct coating morphologies (Figure 1).

The materials were examined by Scanning Electron Microscopy (SEM). In pure SBA-15, i.e., without the use of HAP in any form, we can see layered fiber-like mesoporous material assembled (Figure 1A–D).

**Figure 2:**
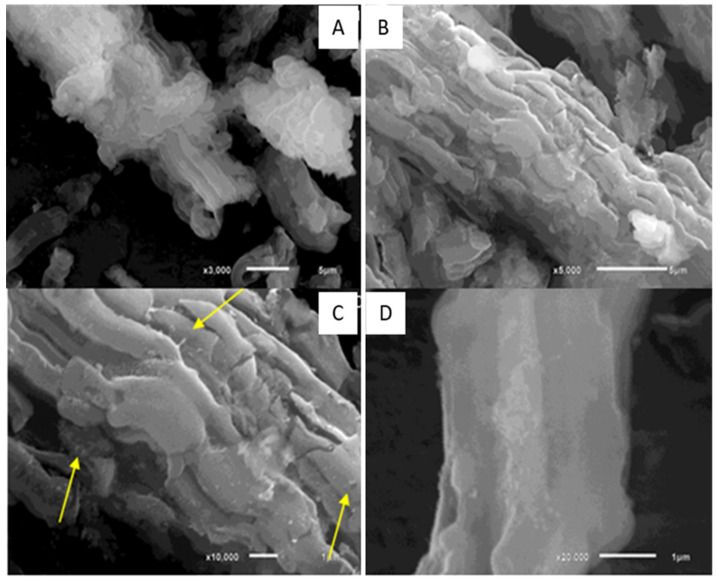
Scanning Electron Microscopy images of the SBA-15/HAP_1 aggregate (**A**,**B**). The yellow arrows (**C**) show the HAP crystals on the mesoporous silica surface, In (**D**) is possible to observe the HAP using a augmented vision (×20,000).

In Figure 2, it is possible to observe that SBA-15/HAP_1 presents changes in its fiber-like structure, both on the surface and in its layers assembly, suggesting growth of HAP crystals not only on the surface but also between the layers.

**Figure 3:**
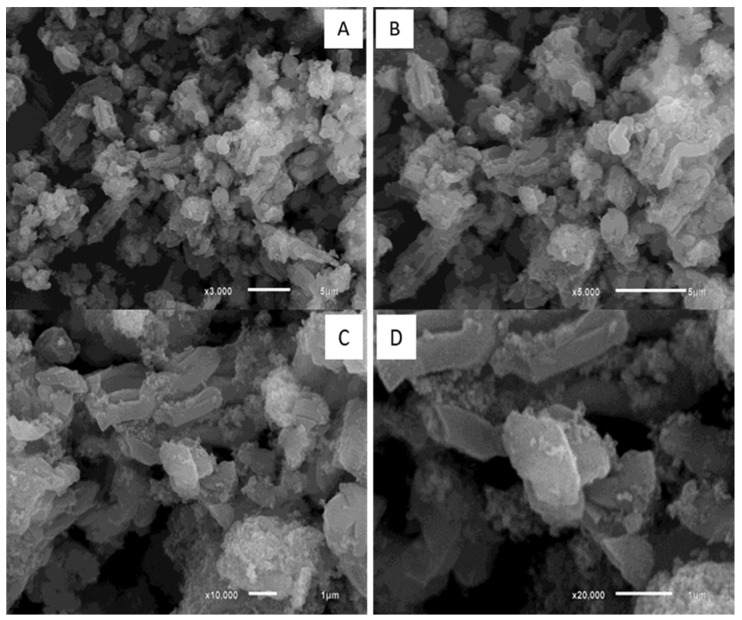
Scanning Electron Microscopy images of SBA-15/HAP_2 structures and their aggregates (**A**–**D**).

Figure 3 shows SEM images of SBA-15/HAP_2 samples. The surface structures present a high amount of HAP crystals. An altered structure of the mesoporous material is observed, losing its fibrillar features, and several terraced surfaces appear, suggesting the increased incorporation of HAP between layers.

**Figure 4:**
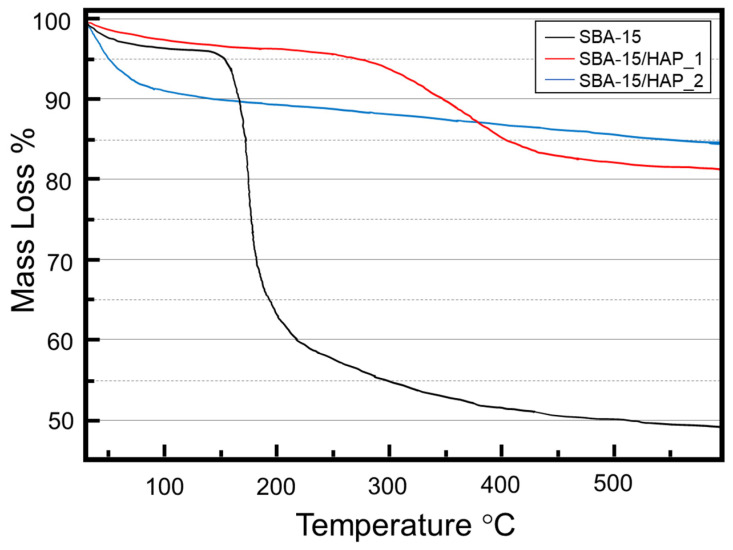
Thermogravimetric profile from SBA-15, SBA-15/HPA_1, and SBA-15/HBA_2 mesoporous silica showing the polymer decomposition as the generation of a mesoporous structure.

The thermogravimetric profile analyses of the non-calcined materials are shown in Figure 4. The decomposition of the copolymer P-123 incorporated in the mesoporous material is observed, and possible species formed during the calcination step. The copolymer P-123 used as a structural leader in the materials synthesis has a boiling point of 250 °C. SBA-15, SBA-15/HAP_1, and SBA-15/HAP_2 samples exhibited small mass losses of 3.6%, 2.8%, and 9.4% at 30–115 °C, respectively. These mass losses are associated with the water elimination adsorbed on the mesoporous material surface, between the layers, and inside the mesopores.

It was observed that the SBA-15/HAP_2 presented a more significant loss of mass associated with adsorbed water compared to SBA-15 and SBA-15/HAP_1. SBA-15 was already calcined as a support for the precipitation of hydroxyapatite was used, which confers a greater surface area to the material and consequent area for the adsorption of water molecules SBA-15/HAP_2. In addition, the spaces between the structures of SBA-15 may have led to higher water adsorption in SBA-15/HAP_2. The sample SBA-15/HAP_2 did not show the thermal degradation of the copolymer P-123 because it was prepared using SBA-15 that was already synthesized and calcined as support.

SBA-15 TGA showed an increased loss of approximately 40% of the mass in a temperature interval of 150–350 °C. This fact is associated with the decomposition and desorption of the copolymer P-123 used in the material synthesis. The result is following the decomposition temperature of 250 °C of the pure copolymer [38]. Similarly, SBA-15/HAP_1 presented loss between 250–400 °C of approximately 13% mass, associated with the decomposition and desorption of the copolymer P-123. This difference in the degradation temperature may be related to the presence of precipitated hydroxyapatite, blocking the mesopores in SBA-15/HAP_1, which may hinder and delay the desorption of the P-123 copolymer outside the mesopores.

### 3.2. Elemental Analysis (EA)

According to the data in Table 1, the Si:Ca:P atomic ratio of 1.00:0.10:0.05 was found for SBA-15/HAP_1. Compared with the Si:Ca:P atomic ratios added of 1.0:1.0:0.6, used in the synthesis, a significant relative decrease in calcium and phosphorus (approximately 10-fold) to the silicon content was observed. The reduction of the contents can be explained by the formation of soluble salts, such as chlorides, phosphates, and silicates, during the material synthesis, which was then solubilized and discarded during the material washing stages. A similar trend was shown for SBA-15/HAP_1.

The amounts of silicon, calcium, and phosphorus (in percentage) in Table 1 corroborate with SEM images, confirming a more significant amount of hydroxyapatite formed in the SBA-15/HAP_2 sample compared to the SBA-15/HAP_1 sample. This fact probably occurs due to the preparation method of the SBA-15/HAP_1, which is carried out in two stages, seeking the precipitation of hydroxyapatite as a priority in the mesopores. The precipitation of hydroxyapatite in mesopores can occur due to the positive potential created by the presence of calcium ions.

The Ca:P atomic ratio of 2.05 was found for SBA-15/HAP_1, revealing calcium ions slightly above the expected value for the stoichiometric hydroxyapatite. According to Elliott, in 1994, calcium-rich apatite, as in the case with a Ca:P ratio higher than 1.7, may be considered mixtures of hydroxyapatite with Ca(OH)_2_ or even apatite-adsorbing calcium ions and an equivalent number of negative ions. However, these types of apatites are formed in methodologies in which temperatures of 1000 °C are employed in ten days. These reported methodologies are distinct from methods used in the materials synthesis. Therefore, this amount of calcium higher than expected for the hydroxyapatite can be explained by the entrapment of calcium ions in the silica matrix, which is expected from obtaining the SBA-15/HAP_1 sample. In contrast, the Ca:P ratio of 1.97 of SBA-15/HAP_2 does not present a statistically significant difference of the Ca:P atomic ratio of 1.94 obtained for HAP due to the synthesis method.

### 3.3. Characterization of the Mesoporous Silica and the Hydroxyapatite Capped Mesoporous Silica Structure

**Figure 5:**
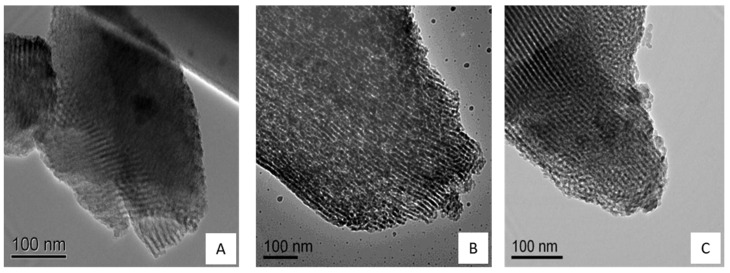
TEM images of mesoporous silica nanoparticles SBA-15 (**A**), SBA-15/HAP_1 (**B**) and SBA-15/HAP_2 (**C**).

The TEM analysis showed some distortions of the 2D hexagonal channels structure on the original silica matrix and HAP clusters around the surface. Figure 5 shows distortions on the more superficial 2D hexagonal channels of the initial silica matrix but not on the inner channels and HAP clusters around the surface. This result suggests that the growth of HAP crystals was not only around the surface but inside the pores of the silica matrix.

### 3.4. Characterization of the Hydroxyapatite Coating of Mesoporous Silica Nanoparticles with Small-Angle X-ray Diffraction (SXRD) and Wide-Angle X-ray Diffraction (WXRD)

**Figure 6:**
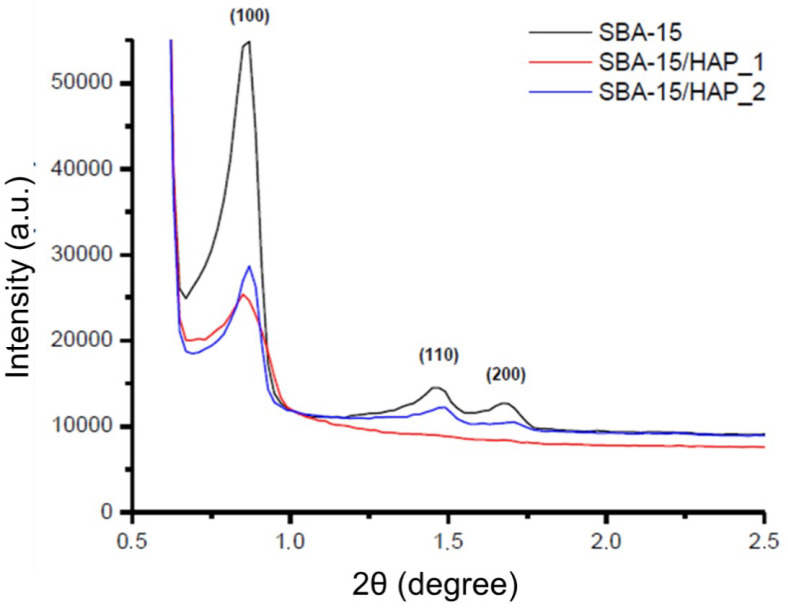
Small-Angle X-ray Diffraction patterns of SBA-15, SBA-15/HAP_1, and SBA-15/HAP_2.

The crystalline structure of the white powdery products was characterized by powder X-ray diffraction (XRD) and Small-angle XRD techniques (Figure 6). SXRD patterns of SBA-15, SBA-15/HAP_1 and SBA-15/HAP_2 exhibit an intense well-defined *d* (100) reflection typical to SBA-15 system mesoporous materials width (100) spacing was 10.19, 10.35, 10.16 A°, respectively. SBA-15 and SBA-15/HAP_2 showed two other resolved peaks, which are indexed as the (110) and (200) reflections with a *d* spacing of 6.04 and 5.25 and 5.96 and 5.17 A°, respectively. These peaks are associated with two-dimensional hexagonal long-range order mesostructured in the space group P6 mm. SBA-15/HAP_1 showed a higher *2θ d* (100) reflection and did not present (110) and (200) reflections, probably because HAP crystallization is inside the pores and Ca ions are inside the silica network.

The Ca ions inside the silica network in SBA-15/HAP_1 can be confirmed by the high amount of Ca verified by elemental analysis compared with SBA-15/HAP_2. According to Morsi and Mohamed [39], the SXRD patterns of SBA mesoporous silica can be corroborated with the display of three peaks at 2*θ*  ≈  2.5, 4.69, and 5.30 A°, which are typical (100), (110), and (200) reflections of one-dimensional hexagonal (P6m) mesostructures, indicating a significant degree of long-range ordering in the structure and a well-formed two-dimensional hexagonal lattice [40].

**Figure 7:**
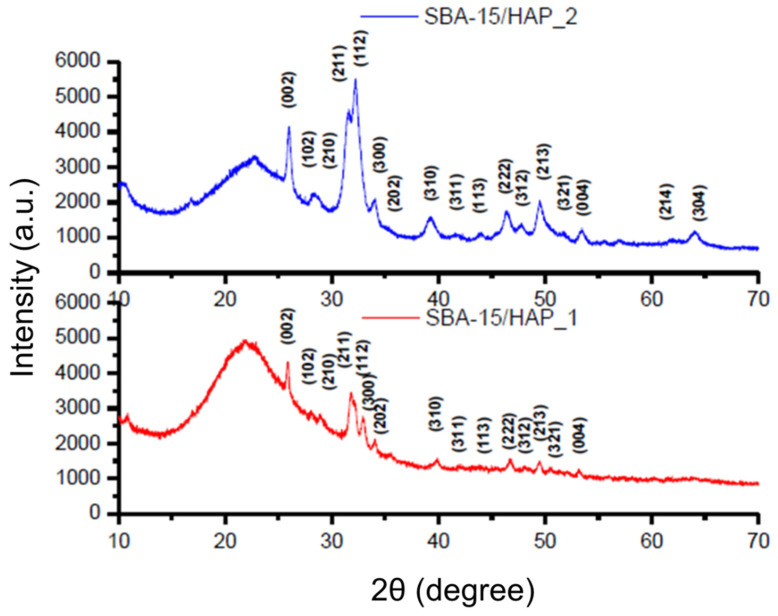
Wide-Angle X-ray Diffraction patterns of SBA-15/HAP_1 and SBA-15/HAP_2.

The wide-angle X-ray diffraction pattern of SBA-15/HAP_1 and SBA-15/HAP_2 indicates the successful formation of HAP crystals as a single crystalline hexagonal phase with space group P6_3_/m in the materials. Characteristic reflections peak positions from planes of the pure HAP phase are indicated in Figure 7. According to Prokopowicz et al. [41] and corroborated by Szewczyk et al. [42], the WXRD analysis of mesoporous silica capped with hydroxyapatite is confirmed with the absence of two peaks at 2*θ*  ≈  4.69 and 5.30, which are typical for (110) and (200) reflections.

All the peaks matched well to those of the HAP phase, and no other peaks were detected. These results likewise reveal a mesoscopic order for the SBA-15 and SBA-15/HAP_2 and the formation of nanocrystals of HAP inside the pores of SBA-15/HAP_1 and SBA-15/HAP_2, which is corroborated by TEM and SEM analysis. The results also demonstrated some defects of the SBA-15/HAP_1 material structure, which showed a similar pattern to the bare SBA-15.

### 3.5. FTIR Analysis

**Figure 8:**
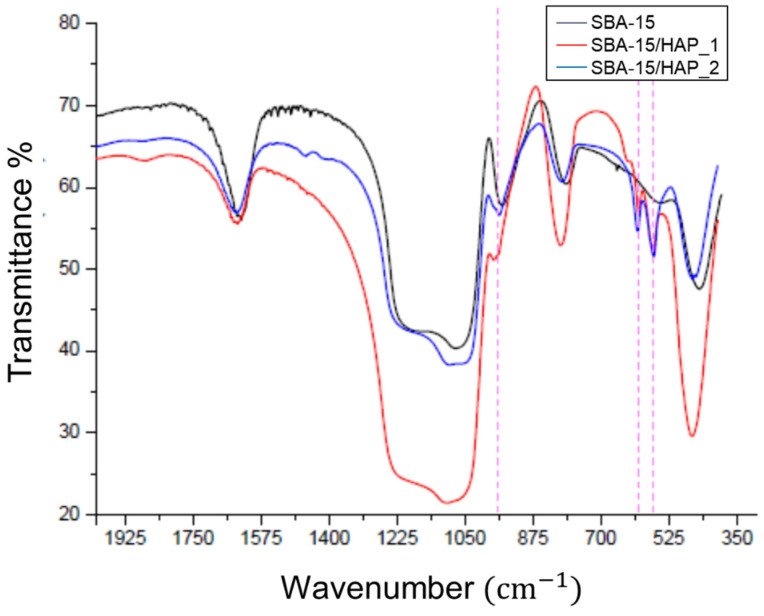
FTIR spectra for calcined SBA-15, SBA-15/HAP_1, and SBA-15/HAP_2 samples.

The FTIR spectra (Figure 8) of SBA-15, SBA-15/HAP_1, and SBA-15/HAP_2 show the Si-OH of silanol groups peak at 960 cm^−1^ presented on the wall of the pores of SBA-15 and SBA-15/HAP_2 but almost disappeared from SBA-15/HAP_1. Therefore, it could be concluded that the Si–OH group, mainly on the pore wall of SBA-15/HAP_1, has been wholly grafted with HAP. In contrast, the Si–OH group is just part of the pores of SBA-15/HAP_2. Both SBA-15/HAP_1 and SBA-15/HAP_2 present two bands at 565 cm^−1^ and 603 cm^−1^ that correspond to the P–O deformation of the phosphate group of HAP. According to Prokopowicz et al. [41] and Ye and Liu Hong [43], the peaks at 603 cm^−1^ and 564 cm^−1^ are due to the (PO_4_)^3−^ groups with the vibrational mode of ν4, whereas the peaks at 1090, 1032, and 961 cm^−1^ were characteristic for the ν3, ν3, and ν1 vibrational modes of (PO_4_)^3−^. The presence of a slight peak at 1090 cm^−1^ in both samples corroborates the existence of CaP [44].

### 3.6. Biological Characterization

Several works have been dedicated to understanding the biocompatibility of nanoparticles/nanosystems with biological systems [45,46,47,48,49]. In the last few years, there has been a growing interest in the use of nanosystems in different biomedical applications, such as targeted drug delivery and disease diagnosis. However, NP’s shape, size, loading capacity, and structure can affect how NPs interact with cells and determine the potential for cytotoxicity. For example, Cho et al. (2018) [48] reported that size-dependent acute toxicity of silver NPs might occur. Additionally, Lai et al. [49] observed cytotoxicity of 10-hydroxycamptothecin (HCPT) nanoparticle dispersions, which depends on the polymorph, in both in vivo and in vitro studies. Finally, Wigner P et al. [45] evaluated the effect of different types of nanoparticles (PLA/MMT/TRASTUZUMAB, PLA/EDTMP, PLGA/MDP, and Pluronic F127 MICELLES) on human endothelial cells and observed, in some cases, high cytotoxicity effect.

### 3.7. Cell Viability—Proliferation

**Figure 9:**
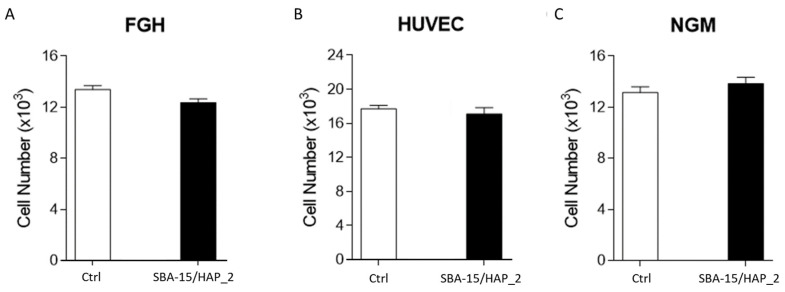
Cytotoxic analysis by MTT of human cells (FGH, HUVEC, and NGM) exposed to SiO_2–_CaO–P_2_O_5_ mesoporous silica for 24 h. In (**A**) is possible to observe no influence on FGH viability. In (**B**) the presence of SBA-150/HAP_2 showed not influenced the HUVEC cells viability the same has been observed in NGM cells (**C**).

The MTT assay test was performed at a 20 ug/mL dose to assess cell viability. The MTT assay showed that when human cells (NGM, FGH, and HUVEC) were exposed to an acute concentration (20 µg/mL) of SBA-15/HAP_2 for 24 h, no significant effect was observed (Figure 9).

The results showed no statistical difference comparing the control and the nanosystem, corroborating that SBA-15/HAP_2 has no cytotoxicity at the dose of 20 µg/mL in HUVEC cells. These data are corroborated by Gonzalez et al. [50]. In their study, they used SBA-15 capped with hydroxyapatite and demonstrated a non-toxic effect on cells. Chen Ying et al. [51] showed that mesoporous silica capped with hydroxyapatite has excellent biocompatibility and may serve as a drug delivery system without influencing cell proliferation. Controversially, Zhao et al. [52], using SBA-15 in three forms (SBA-15, NH2-SBA-15, and COOH-SBA-15), demonstrated that pure SBA-15 is the highest cytotoxic form for HUVEC cells. They also revealed that the carboxyl-modified group (COOH-SBA-15) reduced the cytotoxicity of the SBA-15 by reducing the oxidative stress, and this fact can partially explain the absence of cytotoxicity in the SBA-15/HAP_2 sample.

### 3.8. Immunoblotting

The human cell’s survival depends on the maintenance of intracellular signaling. In this direction, the most important signaling pathways are Akt phosphorylation, which may modulate endothelial cells’ migration, proliferation, and survival. The MAPK/ERK signaling cascade is critical to induce proliferation and differentiation. Finally, glyceraldehyde-3-phosphate dehydrogenase (GAPDH) is related to cellular energy metabolism.

**Figure 10:**
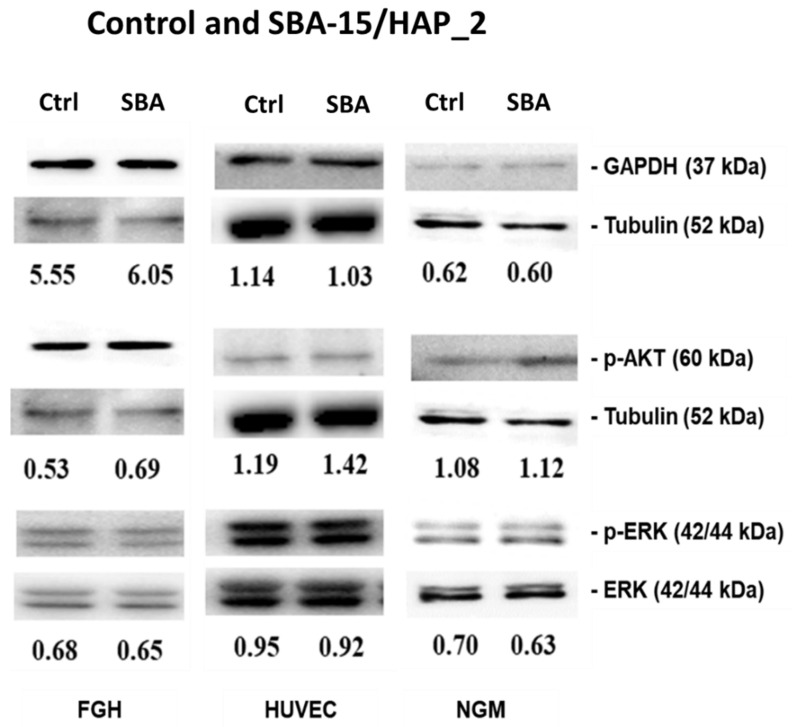
Immunoblotting analysis of human cells exposed to SBA-15/HAP_2 mesoporous silica to evaluate the influence on cell signaling by observing the AKT, p-AKT, ERK, p-ERK, GAPDH, and tubulin.

The immunoblotting results (Figure 10) show that when human cells (NGM, FGH, and HUVEC) are exposed to SBA-15/HAP_2 at a 20 µg/mL concentration, any modification in the expression of Akt and ERK1/2 phosphorylated as GAPDH expression were observed. These data are corroborated by Duan et al. [53]. Their study showed that HUVEC cells showed a dose-dependent manner behavior in the inhibition of MEK1/2 and Akt when exposed to mesoporous silica nanoparticles at a concentration varying from 25-100 µg/mL. Øvrevik et al. [54] have demonstrated that crystalline silica may inhibit the MEK5-ERK5 pathway. In turn, Liu et al. [55] showed that gold-mesoporous silica might influence the cell metabolism by inactivation of ERK. Controversially, Kim et al. [56] demonstrated that mesoporous silica might stimulate the ERK signaling pathway. Sun et al. [57] showed that mesoporous silica nanoparticles did not influence the GAPDH mechanism. Finally, Chauhan et al. [58] demonstrated that mesoporous silica has no significant toxic effect at a low dose, while at the higher doses, toxicity was observed.

## 4. Conclusions

In this work, samples of mesoporous silica capped with hydroxyapatite (SBA-15/HAP_1 and SBA-15/HAP_2) were produced by two methods, making samples with different ultrastructures, different amounts, and various distributions of HAP. The various techniques for characterizing these materials, here employed, revealed that the sample produced by the one-step method (SBA-15/HPA_2) has a significant amount of hydroxyapatite. Therefore, this method is more effective in functionalizing the mesoporous material surface and layers with HAP. For this reason, the SBA-15/HAP_2 sample was used to investigate biocompatibility through cellular and molecular assays, revealing the promising character of this material in biological applications. The results suggest that the insertion of hydroxyapatite in the mesoscopic structure increases its biocompatibility, especially for bone or dental applications.

## Figures and Tables

**Table 1:** Chemical analysis of materials in percentages and atomic ratios Si:Ca: theoretical and experimental P and the atomic ratio Ca:P.

Sample	Si (%)	Ca (%)	P (%)	Atomic Ratio Added Si:Ca:P	Atomic Ratio Founded Si:Ca:P	Atomic Ratio Ca:P
**SBA-15**	38	-	-	-	-	-
**SBA-15/HAP_1**	40	4.1	2	1.0:1.0:0.6	1.00:0.10:0.05	2.05
**SBA-15/HAP_2**	25	12	6.1	1.0:1.0:0.6	1.00:0.48:0.24	1.97
**HAP**	-	35	18	-:1.0:0.6	-:1.00:0.51	1.94

## Data Availability

All raw data will be made available under request.

## References

[1] Nakamura T., Sugihara F., Matsushita H., Yoshioka Y., Mizukami S., Kikuchi K. (2014). Mesoporous silica nanoparticles for 19F magnetic resonance imaging, fluorescence imaging, and drug delivery. Chem. Sci..

[2] Cheng C.-A., Deng T., Lin F.-C., Cai Y., Zink J.I. (2019). Supramolecular Nanomachines as Stimuli-Responsive Gatekeepers on Mesoporous Silica Nanoparticles for Antibiotic and Cancer Drug Delivery. Theranostics.

[3] Thi T.T.H., Nguyen T.N.Q., Hoang D.T., Nguyen D.H. (2019). Functionalized mesoporous silica nanoparticles and biomedical applications. Mater. Sci. Eng. C.

[4] McCarthy C., Ahern R.J., Dontireddy R., Ryan K., Crean A. (2016). Mesoporous silica formulation strategies for drug dissolution enhancement: A review. Expert Opin. Drug Deliv..

[5] Maleki A., Kettiger H., Schoubben A., Rosenholm J.M., Ambrogi V., Hamidi M. (2017). Mesoporous silica materials: From physico-chemical properties to enhanced dissolution of poorly water-soluble drugs. J. Control. Release.

[6] Kumar D., Chirravuri S.S., Shastri N.R. (2014). Impact of surface area of silica particles on dissolution rate and oral bioavailability of poorly water soluble drugs: A case study with aceclofenac. Int. J. Pharm..

[7] Liu H.-J., Xu P. (2019). Smart Mesoporous Silica Nanoparticles for Protein Delivery. Nanomaterials.

[8] Iturrioz-Rodríguez N., Correa-Duarte M.A., Fanarraga M.L. (2019). Controlled drug delivery systems for cancer based on mesoporous silica nanoparticles. Int. J. Nanomed..

[9] Xu C., Lei C., Yu C. (2019). Mesoporous Silica Nanoparticles for Protein Protection and Delivery. Front. Chem..

[10] Lee C.-H., Cheng S.-H., Wang Y.-J., Chen Y.-C., Chen N.-T., Souris J., Chen C.-T., Mou C.-Y., Yang C.-S., Lo L.-W. (2009). Near-Infrared Mesoporous Silica Nanoparticles for Optical Imaging: Characterization and In Vivo Biodistribution. Adv. Funct. Mater..

[11] Cheng S.-H., Li F.-C., Souris J.S., Yang C.-S., Tseng F.-G., Lee H.-S., Chen C.-T., Dong C.-Y., Lo L.-W. (2012). Visualizing Dynamics of Sub-Hepatic Distribution of Nanoparticles Using Intravital Multiphoton Fluorescence Microscopy. ACS Nano.

[12] Slowing I., Trewyn B.G., Lin V.S.-Y. (2006). Effect of Surface Functionalization of MCM-41-Type Mesoporous Silica Nanoparticles on the Endocytosis by Human Cancer Cells. J. Am. Chem. Soc..

[13] Chung T.-H., Wu S.-H., Yao M., Lu C.-W., Lin Y.-S., Hung Y., Mou C.-Y., Chen Y.-C., Huang D.-M. (2007). The effect of surface charge on the uptake and biological function of mesoporous silica nanoparticles in 3T3-L1 cells and human mesenchymal stem cells. Biomaterials.

[14] Lu F., Wu S.-H., Hung Y., Mou C.-Y. (2009). Size Effect on Cell Uptake in Well-Suspended, Uniform Mesoporous Silica Nanoparticles. Small.

[15] Chen Y.-P., Chen H.-A., Hung Y., Chien F.-C., Chen P., Mou C.-Y. (2011). Surface charge effect in intracellular localization of mesoporous silicananoparticles as probed by fluorescent ratiometric pH imaging. RSC Adv..

[16] Chou C.-C., Chen W., Hung Y., Mou C.-Y. (2017). Molecular Elucidation of Biological Response to Mesoporous Silica Nanoparticles in Vitro and in Vivo. ACS Appl. Mater. Interfaces.

[17] Thangaraja A., Savitha V., Jegatheesan K. (2010). Preparation and Characterization of Polyethylene Glycol Coated Silica Nanoparticles for Drug Delivery Application. Int. J. Nanotechnol. Appl..

[18] Cauda V., Argyo C., Bein T. (2010). Impact of different PEGylation patterns on the long-term bio-stability of colloidal mesoporous silica nanoparticles. J. Mater. Chem..

[19] Dos Santos S.N., dos Reis S.R.R., Pires L.P., Helal-Neto E., Sancenon F., Barja-Fidalgo T.C., de Mattos R.M., Nasciutti L.E., Martinez-Manez R., Santos-Oliveira R. (2017). Avoiding the mononuclear phagocyte system using human albumin for mesoporous silica nanoparticle system. Microporous Mesoporous Mater..

[20] Sopyan I., Mel M., Ramesh S., Khalid K.A. (2007). Porous hydroxyapatite for artificial bone applications. Sci. Technol. Adv. Mater..

[21] Hao X., Hu X., Zhang C., Chen S., Li Z., Yang X., Liu H., Jia G., Liu D., Ge K. (2015). Hybrid Mesoporous Silica-Based Drug Carrier Nanostructures with Improved Degradability by Hydroxyapatite. ACS Nano.

[22] Hosseinpour S., Walsh L.J., Xu C. (2020). Biomedical application of mesoporous silica nanoparticles as delivery systems: A biological safety perspective. J. Mater. Chem. B.

[23] Niculescu V.C. (2020). Mesoporous Silica Nanoparticles for Bio-Applications. Front. Mater..

[24] Wang Y., Zhao Q., Han N., Bai L., Li J., Che E., Hu L., Zhang Q., Jiang T., Wang S. (2015). Mesoporous silica nanoparticles in drug delivery and biomedical applications. Nanomed. Nanotechnol. Biol. Med..

[25] Jafari S., Derakhshankhah H., Alaei L., Fattahi A., Varnamkhasti B.S., Saboury A.A. (2019). Mesoporous silica nanoparticles for therapeutic/diagnostic applications. Biomed. pharmacother..

[26] Portilho F.L., Helal-Neto E., Cabezas S.S., Pinto S.R., Dos Santos S.N., Pozzo L., Sancenon F., Martinez-Manez R., Santos-Oliveira R. (2018). Magnetic core mesoporous silica nanoparticles doped with dacarbazine and labelled with 99mTc for early and differential detection of metastatic melanoma by single photon emission computed tomography. Artif. Cells Nanomed. Biotechnol..

[27] Kaliaraj R., Gandhi S., Sundaramurthi D., Sethuraman S., Krishnan U.M. (2018). A biomimetic mesoporous silica–polymer composite scaffold for bone tissue engineering. J. Porous Mater..

[28] Ricci-Junior E., Siqueira L.B.D.O.D., Rodrigues R.A.S., Sancenón F., Martínez-Máñez R., de Moraes J.A., Santos-Oliveira R. (2018). Nanocarriers as phototherapeutic drug delivery system: Appraisal of three different nanosystems in an in vivo and in vitro exploratory study. Photodiagnosis Photodyn. Ther..

[29] Jiang W., Kim B.Y., Rutka J.T., Chan W.C. (2008). Nanoparticle-mediated cellular response is size-dependent. Nat. Nanotechnol..

[30] Alexis F., Pridgen E., Molnar L.K., Farokhzad O.C. (2008). Factors Affecting the Clearance and Biodistribution of Polymeric Nanoparticles. Mol. pharm..

[31] Pasqua L., Cundari S., Ceresa C., Cavaletti G. (2009). Recent Development, Applications, and Perspectives of Mesoporous Silica Particles in Medicine and Biotechnology. Curr. Med. Chem..

[32] Pinto S.R., Helal-Neto E., Paumgartten F., Felzenswalb I., Araujo-Lima C.F., Martínez-Máñez R., Santos-Oliveira R. (2018). Cytotoxicity, genotoxicity, transplacental transfer and tissue disposition in pregnant rats mediated by nanoparticles: The case of magnetic core mesoporous silica nanoparticles. Artif. Cells Nanomed. Biotechnol..

[33] Chen L., Liu J., Zhang Y., Zhang G., Kang Y., Chen A., Feng X., Shao L. (2018). The toxicity of silica nanoparticles to the immune system. Nanomedicine.

[34] Bhavsar D., Patel V., Sawant K. (2019). Systematic investigation of in vitro and in vivo safety, toxicity and degradation of mesoporous silica nanoparticles synthesized using commercial sodium silicate. Microporous Mesoporous Mater..

[35] Díaz A., López T., Manjarrez J., Basaldella E., Martínez-Blanes J., Odriozola J. (2006). Growth of hydroxyapatite in a biocompatible mesoporous ordered silica. Acta Biomater..

[36] Jaffe E.A., Nachman R.L., Becker C.G., Minick C.R. (1973). Culture of human endothelial cells derived from umbilical veins. Identification by morphologic and immunologic criteria. J. Clin. Investig..

[37] Nunes S.S., Outeiro-Bernstein M.A., Juliano L., Vardiero F., Nader H.B., Woods A., Legrand C., Morandi V. (2008). Syndecan-4 contributes to endothelial tubulogenesis through interactions with two motifs inside the pro-angiogenic N-terminal domain of thrombospondin-1. J. Cell. physiol..

[38] Pei L., Kurumada K., Tanigaki M., Hiro M., Susa K. (2005). Effect of drying on the mesoporous structure of sol–gel derived silica with PPO–PEO–PPO template block copolymer. J. Colloid Interface Sci..

[39] Morsi R.E., Mohamed R.S. (2018). Nanostructured mesoporous silica: Influence of the preparation conditions on the physical-surface properties for efficient organic dye uptake. R. Soc. Open Sci..

[40] Awoke Y., Chebude Y., Díaz I. (2020). Controlling Particle Morphology and Pore Size in the Synthesis of Ordered Mesoporous Materials. Molecules.

[41] Prokopowicz M., Szewczyk A., Skwira A., Sadej R., Walker G. (2020). Biphasic composite of calcium phosphate-based mesoporous silica as a novel bone drug delivery system. Drug Deliv. Transl. Res..

[42] Szewczyk A., Skwira A., Ginter M., Tajer D., Prokopowicz M. (2020). Microwave-Assisted Fabrication of Mesoporous Silica-Calcium Phosphate Composites for Dental Application. Polymers.

[43] Ye H., Liu X.Y., Hong H. (2009). Characterization of sintered titanium/hydroxyapatite biocomposite using FTIR spectroscopy. J. Mater. Sci. Mater. Med..

[44] Zhao C.X., Yu L., Middelberg A.P. (2013). Magnetic mesoporous silica nanoparticles end-capped with hydroxyapatite for pH-responsive drug release. J. Mater. Chem. B.

[45] Wigner P., Zielinski K., Michlewska S., Danielska P., Marczak A., Ricci E.J., Santos-Oliveira R., Szwed M. (2021). Disturbance of cellular homeostasis as a molecular risk evaluation of human endothelial cells exposed to nanoparticles. Sci. Rep..

[46] Bhanumathi R., Manivannan M., Thangaraj R., Kannan S. (2018). Drug-Carrying Capacity and Anticancer Effect of the Folic Acid- and Berberine-Loaded Silver Nanomaterial To Regulate the AKT-ERK Pathway in Breast Cancer. ACS Omega.

[47] Shukla R.K., Kumar A., Vallabani N.V.S., Pandey A.K., Dhawan A. (2014). Titanium dioxide nanoparticle-induced oxidative stress triggers DNA damage and hepatic injury in mice. Nanomedicine.

[48] Cho Y.-M., Mizuta Y., Akagi J.I., Toyoda T., Sone M., Ogawa K. (2018). Size-dependent acute toxicity of silver nanoparticles in mice. J. Toxicol. pathol..

[49] Wang Q., Huang J.Y., Li H.Q., Chen Z., Zhao A.Z.J., Wang Y., Zhang K.Q., Sun H.T., Al-Deyab S.S., Lai Y.K. (2016). TiO_2_ nanotube platforms for smart drug delivery: A review. Int. J. Nanomed..

[50] Gonzalez G., Sagarzazu A., Cordova A., Gomes M.E., Salas J., Contreras L., Noris-Suarez K., Lascano L. (2018). Comparative study of two silica mesoporous materials (SBA-16 and SBA-15) modified with a hydroxyapatite layer for clindamycin controlled delivery. Microporous Mesoporous Mater..

[51] Chen Y., Wang J., Zhu X., Fan Y., Zhang X. (2014). Adsorption and Release Behaviors of Vascular Endothelial Growth Factor on Porous Hydroxyapatite Ceramic Under Competitive Conditions. J. Biomater. Tissue Eng..

[52] Zhao J., Bu D., Zhang N., Tian D., Ma L., Yang H. (2021). Cytotoxicity of mesoporous silica modified by amino and carboxyl groups on vascular endothelial cells. Environ. Toxicol..

[53] Duan J., Yu Y., Yü Y., Li Y., Huang P., Zhou X., Peng S., Sun Z. (2014). Silica nanoparticles enhance autophagic activity, disturb endothelial cell homeostasis and impair angiogenesis. Part. Fibre Toxicol..

[54] Øvrevik J., Låg M., Schwarze P., Refsnes M. (2004). p38 and Src-ERK1/2 Pathways Regulate Crystalline Silica-Induced Chemokine Release in Pulmonary Epithelial Cells. Toxicol. Sci..

[55] Tai G., Liu G., Li Q., Ni W., Zhang N., Zheng X., Wang Y., Shao D. (2015). Cytotoxicity of various types of gold-mesoporous silica nanoparticles in human breast cancer cells. Int. J. Nanomed..

[56] Kim K.-A., Kim Y.-H., Seo M.S., Lee W.K., Kim S.W., Kim H., Lee K.-H., Shin I.-C., Han J.-S., Kim H.J. (2002). Mechanism of silica-induced ROS generation in Rat2 fibroblast cells. Toxicol. Lett..

[57] Sun X., Zhang J., Wang Z., Liu B., Zhu S., Zhu L., Peng B. (2019). Licorice isoliquiritigenin-encapsulated mesoporous silica nanoparticles for osteoclast inhibition and bone loss prevention. Theranostics.

[58] Chauhan S., Manivasagam G., Kumar P., Ambasta R.K. (2019). Cellular Toxicity of Mesoporous Silica Nanoparticle in SHSY5Y and BMMNCs Cell. Pharm. Nanotechnol..

